# DREAMM-11, Part 2: Japanese phase I trial of belantamab mafodotin combination therapies in relapsed/refractory multiple myeloma

**DOI:** 10.1007/s12185-024-03889-8

**Published:** 2024-12-24

**Authors:** Kazutaka Sunami, Shinsuke Iida, Nobuhiro Tsukada, Taku Fujii, Hitomi Kato, Ryuichi Fukushima, Satoshi Wakabayashi, Hirofumi Nakano, Sumita Roy-Ghanta, Brandon E. Kremer

**Affiliations:** 1 Department of Hematology, NHO Okayama Medical Center, 1711-1 Tamasu Kitaku, Okayama, Japan; 2 Department of Hematology and Oncology, Nagoya City University Institute of Medical and Pharmaceutical Sciences, Nagoya, Japan; 3 Department of Hematology, Japanese Red Cross Medical Center, Tokyo, Japan; 4 GSK, Tokyo, Japan; 5 Oncology, GSK, Upper Providence, PA USA

**Keywords:** B-cell maturation antigen, Belantamab mafodotin, Japan, Relapsed/refractory multiple myeloma, Combination therapy

## Abstract

DREAMM-11 (NCT03828292) was a Phase 1, open-label, dose-escalation study of belantamab mafodotin in Japanese patients with relapsed/refractory multiple myeloma (RRMM). In Part 1, belantamab mafodotin monotherapy (2.5 or 3.4 mg/kg every 3 weeks) was tolerated and demonstrated clinical activity and a manageable safety profile. Part 2 investigated the tolerability, safety, clinical activity and pharmacokinetics of belantamab mafodotin (2.5 mg/kg on Day [D]1 of each 21-day cycle) plus bortezomib and dexamethasone (Arm A; N = 3) or belantamab mafodotin (2.5 mg/kg on D1 of the first 28-day cycle; 1.9 mg/kg on D1 of subsequent cycles) plus pomalidomide and dexamethasone (Arm B; N = 4) in Japanese patients with RRMM and ≥ 1 prior line of therapy. No dose-limiting toxicities were reported in Arm A; 1 (non-serious liver injury) was reported in Arm B. Safety profiles of each treatment combination were consistent with those of the individual agents and those in Western populations. An overall response was achieved by 3/3 (100%) patients in Arm A and 2/4 (50%) in Arm B. Pharmacokinetics were consistent between Japanese and Western populations. The clinical pharmacokinetics, safety, and efficacy data from this study can inform future use of belantamab mafodotin plus bortezomib/pomalidomide and dexamethasone in Japanese patients with RRMM.

**Supplementary Information:**

The online version contains supplementary material available at 10.1007/s12185-024-03889-8.

## Introduction

Multiple myeloma (MM) is currently incurable and due to the complex molecular pathophysiology of the disease, treatment remains a challenge; most patients eventually relapse and/or become refractory to all available treatments [1]. The substantial symptom burden of MM, in addition to adverse events (AEs) associated with current treatment options, can severely impact a patient’s health-related quality of life (HR-QoL) [2]. Recent advances in the MM treatment landscape have identified B-cell maturation antigen (BCMA) as a tumor-associated antigen and, consequently, the target for multiple immunotherapies including chimeric antigen receptor T-cell (CAR-T) therapy, bispecific/trispecific antibodies (Bs/TsAbs) and antibody–drug conjugates (ADCs) [1].

Belantamab mafodotin is a first-in-class ADC that binds to BCMA on the surface of MM cells, targeting them for elimination via a multimodal mechanism including apoptosis, antibody-dependent cellular cytotoxicity, and phagocytosis [3, 4]. In the Phase 3, open-label trial, DREAMM-3 (NCT04162210), progression-free survival (PFS; 95% confidence interval [CI]) at 12 months was numerically better with belantamab mafodotin monotherapy (48% [40, 56]) compared with pomalidomide plus dexamethasone (35% [23, 48]) [5]. Although PFS superiority of belantamab mafodotin over pomalidomide-dexamethasone (primary endpoint) was not statistically demonstrated, the clinical benefit of belantamab mafodotin monotherapy was sustained in patients who responded to treatment, and the safety profile was manageable (consistent with that reported in the Phase 2 trial, DREAMM-2) [5, 6]. More recently, clinical studies have explored the efficacy and safety of combinations of belantamab mafodotin with standard of care (SoC) regimens such as bortezomib plus dexamethasone (DREAMM-7; NCT04246047) and pomalidomide plus dexamethasone (ALGONQUIN; NCT03715478 and DREAMM-8; NCT04484623) [7, 8]. Clinical activity of belantamab mafodotin was demonstrated in the ALGONQUIN trial, which reported a median PFS of 21.8 months (95% CI 17.8, 24.2) across all dose cohorts investigated, and demonstrated a manageable safety profile that helped to inform on dosing for the currently ongoing DREAMM-8 trial [8, 9]. Median PFS had not been reached for patients treated with belantamab mafodotin and pomalidomide plus dexamethasone in DREAMM-8 while the control arm had a median PFS of 12.7 months (P < 0.001) [9]. DREAMM-7 reported a statistically significant PFS benefit (36.6 months vs 13.4 months) and a clinically meaningful OS trend for belantamab mafodotin versus the SoC regimen, daratumumab plus bortezomib and dexamethasone, as well as a manageable safety profile that was consistent with that of the individual agents [10].

To date, studies of belantamab mafodotin in RRMM have primarily been conducted in Western patient populations. DREAMM-11, a Phase 1, open-label, multicenter, dose-escalation study, was conducted to assess the tolerability, safety, efficacy and pharmacokinetics (PK) of belantamab mafodotin in Japanese patients with RRMM, potentially providing support for the investigation of belantamab mafodotin in this patient population in ongoing global Phase 3 trials [11]. In Part 1 of DREAMM-11, belantamab mafodotin monotherapy (2.5 mg/kg or 3.4 mg/kg every 3 weeks [Q3W]) was tolerated, had a manageable safety profile and demonstrated evidence of clinical activity [11]. No major differences in PK or safety profile were observed between the Japanese patient population and the Western patient populations previously investigated [11].

This manuscript reports the results of Part 2 of DREAMM-11, which investigated the tolerability, safety, efficacy and PK of belantamab mafodotin in combination with the approved regimens, bortezomib plus dexamethasone and pomalidomide plus dexamethasone, in Japanese patients with RRMM.

## Materials and methods

### Patients

Full details of the patient eligibility criteria for this study are listed in the Supplementary Materials, Sect.  1. Briefly, eligible patients were ≥ 20 years of age with a confirmed MM diagnosis and an Eastern Cooperative Oncology Group performance status of 0–2. All eligible patients had undergone autologous stem-cell transplant > 100 days prior to study enrolment or were transplant ineligible. Eligible patients had also received ≥ 1 prior line of antimyeloma therapy, with disease progression recorded during or after their most recent therapy. Patients were excluded if they had received prior BCMA-targeted therapy or allogeneic stem cell transplant, had received monoclonal antibody (mAb) therapy ≤ 30 days prior to enrolment, or had current corneal epithelial disease (except for mild punctate keratopathy). Additional exclusion criteria for Arm A included intolerance/refractoriness to bortezomib, ongoing Grade ≥ 2 peripheral neuropathy or neuropathic pain, and intolerance or contraindications to herpes zoster prophylaxis. Additional exclusion criteria for Arm B included prior pomalidomide use, intolerance or contraindications to anti-thrombotic prophylaxis, and active or a history of venous thromboembolism ≤ 3 months prior to first dose of study intervention.

### Study design

DREAMM-11 Part 2 (NCT03828292) was a Phase 1, open-label trial to investigate the tolerability, safety, clinical activity, PK, pharmacodynamics (PD) and immunogenicity of belantamab mafodotin in combination with bortezomib or pomalidomide plus dexamethasone. In Arm A, patients received intravenous (IV) belantamab mafodotin (2.5 mg/kg Q3W on Day 1 of each 21-day treatment cycle), subcutaneous (SC) bortezomib (1.3 mg/m^2^ on Days 1, 4, 8 and 11 of each cycle, Q3W) and oral dexamethasone (20 mg on Days 1, 2, 4, 5, 8, 9, 11 and 12 of each cycle, Q3W). Patients in Arm A received bortezomib and dexamethasone for a total of 8 cycles and continued to receive belantamab mafodotin (2.5 mg/kg) until disease progression, withdrawal of consent or unacceptable toxicity were reached. In Arm B, patients received IV belantamab mafodotin (2.5 mg/kg on Day 1 of a single 28-day cycle, and 1.9 mg/kg on Day 1 of each subsequent 28-day cycle), oral pomalidomide (4 mg daily on Days 1–21 of each cycle, Q4W) and oral dexamethasone (40 mg on Days 1, 8, 15 and 22 of each cycle, Q4W). Patients in Arm B received belantamab mafodotin, pomalidomide and dexamethasone until disease progression, withdrawal of consent or unacceptable toxicity were reached (Supplementary Fig. 1). At the time of data cut for final analysis (06 April 2023), any patients who were still benefiting from belantamab mafodotin were permitted to continue the treatment.

## Endpoints and assessments

### Tolerability and safety

To assess the tolerability and safety of belantamab mafodotin, the primary endpoints of DREAMM-11 Part 2 were dose-limiting toxicities (DLTs), AEs, and changes in clinical signs and parameters reported during the study period (up to 70 days post-last dose of study intervention). Only key safety data are reported for those patients who were permitted to continue treatment after the final analysis. AEs were graded according to the National Cancer Institute Common Terminology Criteria for Adverse Events (NCI-CTCAE) and corneal events were graded according to a protocol-defined scale for keratopathy and visual acuity that captures both corneal findings and visual acuity changes (Supplementary Table 1). Any AEs observed in the first 21 days (Arm A) or 28 days (Arm B) of the treatment cycle were considered DLTs if a relationship to the treatment agent could not be ruled out. Protocol-defined DLT events included Grade ≥ 3 febrile neutropenia lasting > 48 h, Grade 4 thrombocytopenia with clinically significant bleeding, Grade ≥ 3 non-hematologic toxicity (other than corneal events) for > 48 h, Grade ≥ 3 non-hematologic laboratory value for > 48 h or requiring hospitalization, Grade 4 corneal events and liver toxicity meeting stopping criteria. Protocol-defined AEs of special interest (AESI) were ocular events, thrombocytopenia and infusion-related reactions (IRRs). Prophylaxis for corneal events was administered, including preservative-free artificial tears administered daily from the start of Cycle 1, and optional use of a cooling eye mask during belantamab mafodotin infusion. To protect the ethical and safety interests of participants, as well as the scientific validity of the study, a Safety and Efficacy Evaluation Committee (SEEC) was employed to medically or statistically review safety and/or efficacy issues and provide recommendations throughout the study.

### Efficacy

The clinical activity of belantamab mafodotin was evaluated as a secondary objective per the International Myeloma Working Group Response Criteria [12]. Secondary endpoints in DREAMM-11 Part 2 included overall response rate (ORR), defined as the percentage of patients achieving a confirmed partial response (PR) or better, and clinical benefit rate (CBR), defined as the percentage of patients with a minimal response (MR) or better.

### Pharmacokinetics and immunogenicity

PK and immunogenicity were investigated as secondary objectives. PK parameters of the belantamab mafodotin ADC and total antibody (sum of ADC and naked mAb without cysteine maleimidocaproyl monomethyl auristatin F [cys-mcMMAF], the microtubule inhibitor released from belantamab mafodotin during antibody proteolysis) and cys-mcMMAF were assessed. For each analyte, these included maximum observed concentration (C_max_), time to C_max_ (t_max_), the last time point where the analyte concentration exceeded the limit of quantification (t_last_) and the area under the concentration–time curve (AUC) up to last quantifiable study time-point (AUC_(0-tlast)_) assessed after a single dose. For belantamab mafodotin ADC and total antibody only, concentration at end of infusion (C_EOI_) was assessed after repeat dose, while AUC for one dosing interval (AUC_(0-tau)_), AUC from zero to infinity (AUC_(0-inf)_), clearance (CL), volume of distribution at steady state (Vss) and terminal phase half-life (t_½_) were assessed after single dose. Incidence and titers of anti-drug antibodies were also assessed.

## Statistical analysis

The primary analyses were conducted per treatment arm by the data cut-off of 06 April 2023, after end of treatment (EOT) and the completion of AE and SAE data collections. Based on the 3 + 3 design, a maximum of 6 patients was required for each arm of the study. The all treated population, comprising eligible patients who received ≥ 1 dose of study intervention, was used for safety and efficacy analyses. Patients who received a complete infusion of belantamab mafodotin and at least 75% of planned doses of bortezomib plus dexamethasone (Arm A) or pomalidomide plus dexamethasone (Arm B) by the end of Cycle 1 were considered DLT evaluable. Patients in the all treated population from whom ≥ 1 PK sample was obtained and analyzed were included in the PK population. Patients in the all treated population from whom ≥ 1 PD sample was obtained, analyzed and was measurable were included in the PD population.

## Results

### Patient disposition

A total of three patients were screened and enrolled in Arm A, two of which completed the study; one patient discontinued belantamab mafodotin (due to study withdrawal by patient, associated with the patient experiencing anxiety) before the data cut-off date. All three patients in Arm A completed the 8-cycle bortezomib plus dexamethasone treatment as planned, and were therefore included in both the all treated and DLT Evaluable populations. Two patients continued to receive belantamab mafodotin treatment up to and beyond the data cut-off date. A total of four patients were screened and enrolled in Arm B, all of which completed the study and were therefore included in both the all treated and DLT Evaluable populations. As of the data cut-off date, two patients in Arm B had discontinued all study treatments: one due to disease progression, and one due to an AE. The remaining two patients received belantamab mafodotin treatment up to and beyond the data cut-off date. One patient discontinued pomalidomide treatment as of the data cut-off date, due to protocol-defined stopping criteria. No patients died during the study.

### Patient demographics and clinical characteristics

Patient demographics and clinical characteristics are presented in Table 1**.** In Arm A, the mean age was 70.0 (standard deviation [SD] 5.2) years. All patients had International Staging System (ISS) Stage I MM and had received 4–7 prior lines of therapy. No patients had evidence of extramedullary disease (EMD), and two patients had high-risk cytogenetics. In Arm B, the mean age was 61.8 (SD 14.9) years. Three patients had ISS Stage I MM and one had ISS Stage II MM. All patients had received 1–4 prior lines of therapy. No patients had evidence of EMD; one patient had high-risk cytogenetics.

**Table 1:** Patient demographics and baseline characteristics (all treated population)

	Arm A Belantamab mafodotin + bortezomib/dexamethasone (N = 3)	Arm B Belantamab mafodotin + pomalidomide/dexamethasone (N = 4)
Patient demographics		
Age, mean years (SD)	70.0 (5.2)	61.8 (14.9)
Baseline disease characteristics		
ISS stage, n (%)		
I	3 (100)	3 (75)
II	0 (0)	1 (25)
R-ISS stage, n (%)		
I	1 (33)	2 (50)
II	2 (67)	2 (50)
Prior lines of therapy, n (%)		
1 line	0 (0)	1 (25)
2 lines	0 (0)	2 (50)
4 lines	1 (33)	1 (25)
5 lines	1 (33)	0 (0)
7 lines	1 (33)	0 (0)
Type of MM, n (%)		
Secretory	3 (100)	4 (100)
Myeloma light chain, n (%)		
Kappa light chain	1 (33)	4 (100)
Lambda light chain	1 (33)	0 (0)
Missing	1 (33)	0 (0)
Myeloma immunoglobulin, n (%)		
IgA	3 (100)	0 (0)
IgG	0 (0)	3 (75)
BJP	0 (0)	1 (25)
Extramedullary disease, n (%)		
No	3 (100)	4 (100)
Lytic bone lesions, n (%)		
Yes	2 (67)	4 (100)
No	1 (33)	0 (0)
Cytogenetic risk, n (%)		
High risk	2 (67)^a^	1 (25)^b^
Other (non-high risk, not done or missing)	1 (33)	3 (75)
Prior anti-cancer therapy^c^, n (%)		
Steroids	3 (100)	4 (100)
Immunomodulator	3 (100)	3 (75)
Lenalidomide	3 (100)	3 (75)
Pomalidomide	2 (67)	0 (0)
Thalidomide	1 (33)	0 (0)
Proteasome inhibitor	3 (100)	4 (100)
Bortezomib	3 (100)	4 (100)
Carfilzomib	2 (67)	0 (0)
Ixazomib	2 (67)	1 (25)
Monoclonal antibody	2 (67)	1 (25)
Elotuzumab	2 (67)	1 (25)
HDAC inhibitor	1 (33)	0 (0)
Panobinostat	1 (33)	0 (0)
Chemotherapy	3 (100)	4 (100)
Other (blinded trial medication)	1 (33)	1 (25)

*BJP* Bence Jones protein, *HDAC* histone deacetylase, *Ig* immunoglobulin, *ISS* International Staging System, *MM* multiple myeloma, *R-ISS* Revised ISS, *SD* standard deviation

^a^One patient had a positive result for t(14;16) and one had a positive result for t(14;16) and 17p13del

^b^Patient had a positive result for t(4;14)

^c^Multiple categories possible per patient, total may be > 100%. No patients had ISS Stage III or non-secretory MM, and no patients had IgD, IgE or IgM myeloma immunoglobulin

## Exposure

### Treatment

Exposure to study treatment is summarized in Table 2**.** The median dose intensity was 0.386 (range: 0.34–1.46) mg/kg/3 weeks in Arm A and 1.500 (range: 0.50–2.50) mg/kg/4 weeks in Arm B (0.5 mg/kg/4 weeks for the two patients who achieved an overall response in Arm B). Median relative dose intensities were 15.43% (range: 13.40–58.33) in Arm A and 62.99% (range: 25.97–100.00) in Arm B. The median duration of exposure was 90.4 weeks and 50.0 weeks in Arms A and B, respectively. Two patients in each arm continued to receive belantamab mafodotin post data cut-off; the on-treatment period ranged from 99.6 to 123 weeks in Arm A and from 100.1 to 104.1 weeks in Arm B.

**Table 2:** Belantamab mafodotin treatment exposure (all treated population)

	Arm A Belantamab mafodotin + bortezomib/dexamethasone (N = 3)	Arm B Belantamab mafodotin + pomalidomide/dexamethasone (N = 4)
Dose intensity (Arm A: mg/kg Q3W; Arm B: mg/kg Q4W)^a^		
Mean (SD)	0.726 (0.6344)	1.500 (1.1547)
Median (range)	0.386 (0.34–1.46)	1.500 (0.50–2.50)
Relative dose intensity, %^b^		
Mean (SD)	29.06 (25.38)	62.99 (42.74)
Median (range)	15.43 (13.40–58.33)	62.99 (25.97–100.00)
Number of cycles^c^		
Mean (SD)	5.3 (2.52)	3.5 (2.89)
Median (range)	5.0 (3–8)	3.5 (1–6)
≤ 4 cycles, n (%)	1 (33)	2 (50)
> 4 cycles, n (%)	2 (67)	2 (50)
Duration of exposure (weeks)		
Mean (SD)	76.2 (55.10)	50.0 (53.12)
Median (range)	90.4 (15–123)	50.0 (4–96)
Cumulative actual dose^d^ (mg/kg)		
Mean (SD)	11.133 (4.2454)	7.250 (5.4848)
Median (range)	10.100 (7.50–15.80)	7.250 (2.50–12.00)

*Q#W* every # weeks, *SD* standard deviation

^a^Arm A intensity = Cumulative actual dose/(last dose date minus first dose date + 21/21), Arm B intensity = Cumulative actual dose/(last dose date minus first dose date + 28/28)

^b^Relative dose intensity = 100*(overall dose intensity/planned dose intensity of 2.5 mg/kg)

^c^Cycles during which dose was given

^d^Sum of the dose at each cycle

### Belantamab mafodotin dose modifications

**Fig. 1:**
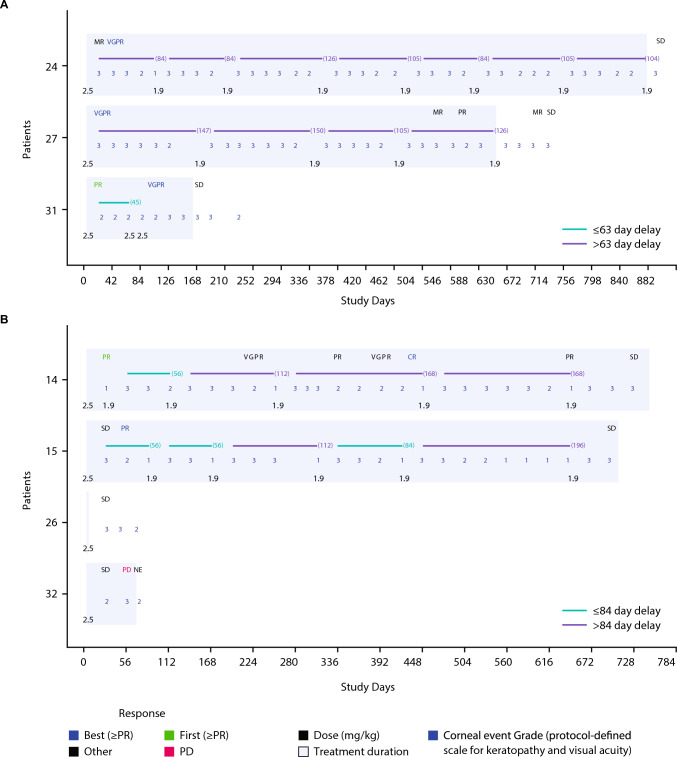
Profile plots of belantamab mafodotin and response in **A** Arm A and **B** Arm B (all treated population). This figure was adapted from Iida S, et al. 49th Annual Meeting of the Japanese Society of Myeloma, May 31–June 1, 2024 and has been reused with permission. *CR* complete response, *MR* minimal response, *NE* not evaluable, *PD* progressive disease, *PR* partial response, *SD* stable disease, *VGPR* very good partial response

In Arm A, two patients (67%) required dose reductions due to Grade 3 corneal events; due to the pre-planned reduction in dose after Cycle 1 from 2.5 mg/kg to 1.9 mg/kg, no dose reductions were permitted in Arm B. All three patients (100%) in Arm A required a total of 12 dose delays; all of which lasted more than 42 days and were due to corneal events. In Arm B, two patients (50%) required a total of 9 dose delays; 7 (78%) were due to corneal events and 2 (22%) were due to other AEs. Three of the dose delays lasted 29–56 days, while the remaining 6 dose delays lasted > 56 days (Supplementary Table 2 and Fig. 1).

## Tolerability and safety

### Dose-limiting toxicities

No DLTs were reported in Arm A, while one DLT of non-serious Grade 3 liver injury (indicated by elevated alanine aminotransferase, aspartate aminotransferase, alkaline phosphatase and gamma glutamyl transferase) was reported during Cycle 1 of Arm B. This DLT was considered related to the combination of belantamab mafodotin plus pomalidomide and dexamethasone, and was resolved following discontinuation of all study interventions; this patient was still included in the safety and efficacy assessments.

### Adverse events

All three (100%) patients in Arm A and four (100%) patients in Arm B experienced ≥ 1 AE related to belantamab mafodotin. All patients experienced non-serious Grade ≥ 3 AEs, the most common being thrombocytopenia (n = 3 [100%] patients; two of which were Grade 4) and lymphopenia (n = 2 [67%] patients) in Arm A and decreased neutrophil count (n = 2 [50%] patients; both Grade 4) and thrombocytopenia (n = 2 [50%] patients; one of which was Grade 4) in Arm B (Table 3). No patients in Arm A experienced an SAE; one (25%) patient in Arm B experienced an SAE of Grade 2 coronavirus disease 2019 (COVID-19) which was not deemed to be related to the study interventions and was resolved with supportive treatment and delay/interruption of study treatments. There were no fatal AEs in either treatment arm.

**Table 3:** Adverse events of all grades by preferred term (all treated population)

	Arm A Belantamab mafodotin + bortezomib/dexamethasone (N = 3)		Arm B Belantamab mafodotin + pomalidomide/dexamethasone (N = 4)	
	Any grade	Grade ≥ 3	Any grade	Grade ≥ 3
Any events, n (%)	3 (100)	3 (100)	4 (100)	4 (100)
Hematologic events				
Thrombocytopenia^a^	3 (100)	3 (100)	4 (100)	4 (100)
Lymphopenia	2 (67)	2 (67)	1 (25)	1 (25)
Neutrophil count decreased	0 (0)	0 (0)	2 (50)	2 (50)
Anemia	0 (0)	0 (0)	1 (25)	1 (25)
Iron deficiency anemia	1 (33)	1 (33)	0 (0)	0 (0)
Non-hematologic events				
Insomnia	2 (67)	1 (33)	1 (25)	0 (0)
Constipation	0 (0)	0 (0)	2 (50)	0 (0)
Diarrhea	2 (67)	1 (33)	0 (0)	0 (0)
Peripheral edema	0 (0)	0 (0)	2 (50)	0 (0)
Peripheral sensory neuropathy	2 (67)	0 (0)	0 (0)	0 (0)
Abnormal feces	0 (0)	0 (0)	1 (25)	0 (0)
ALT increased	0 (0)	0 (0)	1 (25)	0 (0)
Amylase increased	0 (0)	0 (0)	1 (25)	1 (25)
Arthritis	1 (33)	0 (0)	0 (0)	0 (0)
Asteatosis	0 (0)	0 (0)	1 (25)	0 (0)
AST increased	0 (0)	0 (0)	1 (25)	0 (0)
Back pain	1 (33)	0 (0)	0 (0)	0 (0)
Blood phosphorous decreased	0 (0)	0 (0)	1 (25)	1 (25)
COVID-19	0 (0)	0 (0)	1 (25)	0 (0)
Candida infection	0 (0)	0 (0)	1 (25)	0 (0)
Cataract	0 (0)	0 (0)	1 (25)	0 (0)
Dermatophytosis of nail	0 (0)	0 (0)	1 (25)	0 (0)
Feces soft	0 (0)	0 (0)	1 (25)	0 (0)
Fall	0 (0)	0 (0)	1 (25)	0 (0)
Hepatic function abnormal	0 (0)	0 (0)	1 (25)	0 (0)
Hiccups	1 (33)	0 (0)	1 (25)	0 (0)
Hyperglycemia	0 (0)	0 (0)	1 (25)	0 (0)
Hypertension	1 (33)	1 (33)	0 (0)	0 (0)
Hypoacusis	0 (0)	0 (0)	1 (25)	0 (0)
Hypokalemia	1 (33)	1 (33)	0 (0)	0 (0)
Ingrowing nail	0 (0)	0 (0)	1 (25)	0 (0)
Liver injury	0 (0)	0 (0)	1 (25)	1 (25)
Nasopharyngitis	0 (0)	0 (0)	1 (25)	0 (0)
Nausea	0 (0)	0 (0)	1 (25)	0 (0)
Oral mucosa erosion	0 (0)	0 (0)	1 (25)	0 (0)
Peripheral neuropathy	0 (0)	0 (0)	1 (25)	0 (0)
Periodontitis	0 (0)	0 (0)	1 (25)	0 (0)
Plantar fasciitis	0 (0)	0 (0)	1 (25)	0 (0)
Pruritus	0 (0)	0 (0)	1 (25)	0 (0)
Rash	0 (0)	0 (0)	1 (25)	0 (0)
Seborrheic keratosis	0 (0)	0 (0)	1 (25)	0 (0)
Stomatitis	0 (0)	0 (0)	1 (25)	0 (0)
Tinea nigra	1 (33)	0 (0)	0 (0)	0 (0)
Tongue hemorrhage	0 (0)	0 (0)	1 (25)	0 (0)
Upper abdominal pain	1 (33)	0 (0)	0 (0)	0 (0)

*AESI* adverse event of special interest, *ALT* alanine aminotransferase, *AST* aspartate aminotransferase, *COVID-19* coronavirus disease 2019, *NCI-CTCAE* National Cancer Institute Common Terminology Criteria for Adverse Events

^a^AESI, include thrombocytopenia and platelet count decreased. No ocular AESIs by NCI-CTCAE were reported

### Adverse events of special interest

No ocular AESIs according to the NCI-CTCAE were reported for either treatment arm. However, based on the protocol-defined scale for keratopathy and visual acuity (Supplementary Table 1), corneal events were reported in three (100%) and four (100%) patients in Arm A and Arm B, respectively; the maximum Grade of all corneal events was Grade 3. In Arm A, onset of the first Grade ≥ 2 corneal event occurred within 22–42 days of treatment for all patients; they were managed by dose reductions in 2 patients and dose delays/interruptions in all 3 patients and were resolved after a median of 127 days, with no treatment withdrawal required (Supplementary Table 3). In Arm B, onset of the first Grade ≥ 2 corneal event occurred within 29–56 days of treatment for three patients and within 57–84 days for one patient. All Grade 3 corneal events were managed by dose delays/interruptions, with one patient requiring a dose delay when the corneal event was Grade 2. Treatment withdrawal was not required for any patient, and the events were resolved after a median of 78 days (Supplementary Table 3).

All three patients in Arm A had ≥ 1 thrombocytopenic event, with Grade 3 thrombocytopenia reported for 1 patient, and Grade 4 thrombocytopenia reported for two patients. These events required bortezomib dose reduction and delay in two patients. At the time of data cut-off, thrombocytopenia had resolved in one patient. All four patients in Arm B had ≥ 1 thrombocytopenic event (either decreased platelet count or thrombocytopenia); with a maximum of Grade 2 for two patients, and Grades 3 and 4 for one patient each. One patient required dose reduction and delay of pomalidomide. At the time of data cut-off, all thrombocytopenic events had resolved. No patients experienced an IRR related to belantamab mafodotin in either treatment arm.

### Efficacy

The ORR in Arm A (belantamab mafodotin plus bortezomib and dexamethasone) and Arm B (belantamab mafodotin plus pomalidomide and dexamethasone) was 100% and 50%, respectively (Table 4 and Fig. 1). All patients in Arm A achieved a VGPR. One patient in Arm B achieved a CR, and another patient achieved a PR. The CBR (95% CI) was 100% (29.2, 100.0) and 50% (6.8, 93.2) in Arm A and Arm B, respectively. Two of the responders in Arm A and one of the responders in Arm B had high-risk cytogenetics. Two of the three responders in Arm A and both of the responders in Arm B had a long treatment duration (over 600 days).

**Table 4:** Overall response and clinical benefit rates (all treated population)

	Arm A Belantamab mafodotin + bortezomib/dexamethasone (N = 3)	Arm B Belantamab mafodotin + pomalidomide/dexamethasone (N = 4)
Best response, n (%)		
sCR	0 (0)	0 (0)
CR	0 (0)	1 (25)
VGPR	3 (100)	0 (0)
PR	0 (0)	1 (25)
MR	0 (0)	0 (0)
SD	0 (0)	2 (50)
ORR, % (95% CI^a^)	100 (29.2, 100.0)	50 (6.8, 93.2)
CBR, % (95% CI^a^)	100 (29.2, 100.0)	50 (6.8, 93.2)

*CBR* clinical benefit rate, *CI* confidence interval, *CR* complete response, *MR* minimal response, *ORR* overall response rate, *PR* partial response, *sCR* stringent complete response, *SD* stable disease, *VGPR* very good partial response

^a^Based on Exact method

### Pharmacokinetics and immunogenicity

**Fig. 2:**
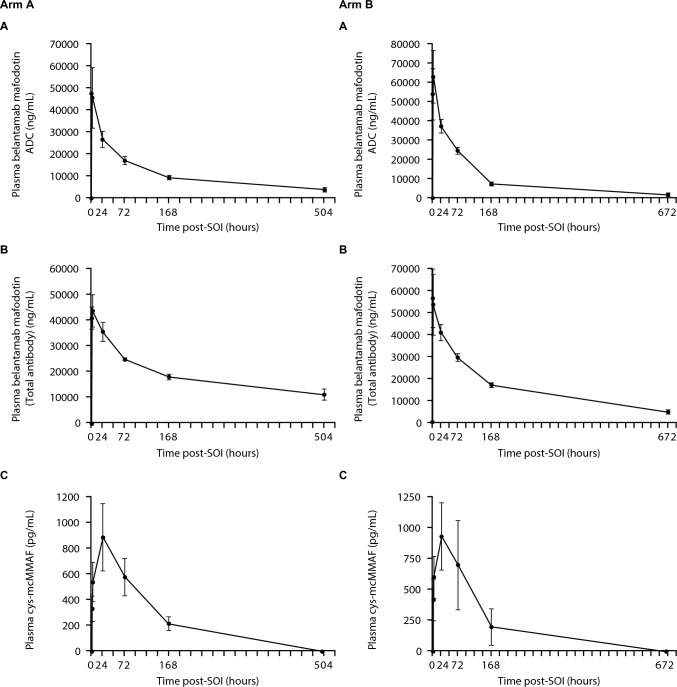
Median plasma concentrations of **A** belantamab mafodotin ADC, **B** total antibody, and **C** cys-mcMMAF over time during Cycle 1, for Arm A (left panel) and Arm B (right panel) (PK population). *ADC* antibody–drug conjugate, *cys-mcMMAF* cysteine maleimidocaproyl monomethyl auristatin F, *SOI* start of infusion

The PK parameters for the belantamab mafodotin ADC and total antibody, and for cys-mcMMAF for Cycle 1 of each treatment arm were similar between the two cohorts (summarized in Tables 5 and 6). While peak plasma concentrations were observed close to the end of the infusion for ADC and total antibody, for cys-mcMMAF peak plasma concentrations occurred almost 24 h post-dose (Fig. 2). The geometric mean AUC_(0–tau)_ for ADC and total antibody was 5077.3 h*μg/mL and 9213.7 h*μg/mL, respectively, for Arm A and 5489.7 h*μg/mL and 9590.9 h*μg/mL for Arm B. The geometric mean AUC_(0–tlast)_ for cys-mcMMAF was 80.1 h*ng/mL in Arm A and 82.6 h*ng/mL in Arm B. All seven patients were negative for anti-belantamab mafodotin antibodies at baseline and post baseline.

**Table 5:** Summary of derived belantamab mafodotin PK parameters during Cycle 1 (PK population)

Geometric mean (% CVb)	Arm A Belantamab mafodotin + pomalidomide/dexamethasone (N = 3)		Arm B Belantamab mafodotin + pomalidomide/dexamethasone (N = 4)	
	ADC	Total antibody	ADC	Total antibody
C_EOI_ (μg/mL)	46.3 (27.1)	40.8 (11.0)	53.0 (21.3)	55.9 (12.6)
C_max_ (μg/mL)	47.6 (25.1)	43.5 (15.1)	61.7 (26.5)	52.3 (22.6)
t_max_ (h^a^)	0.8 (0.7–1.9)	1.9 (1.8–2.2)	1.9 (0.8–2.2)	1.9 (1.8–2.2)
t_last_ (day^a^)	21.1 (21.0–24.1)	21.1 (21.0–24.1)	28.0 (6.9–28.0)	28.0 (6.9–28.0)
AUC_(0-tlast)_ (h*μg/mL)	5160.3 (10.8)	9472.0 (2.0)	5166.5 (31.9)	8495.8 (36.7)
AUC_(0-tau)_ (h*μg/mL)	5077.3 (13.1)	9213.7 (6.5)	5489.7 (25.4)	9590.9 (23.7)
AUC_(0-inf)_ (h*μg/mL)	5442.8; 7066.6^b^	–	6178.4 (30.8)	9146.2; 10,763.3^b^
CL (mL/h)	23.9; 25.5^b^	–	20.9 (7.2)	10.8; 13.9^b^
Vss (L)	6.6; 7.0^b^	–	4.5 (22.0)	4.1; 4.7^b^
t_½_ (day)^c^	9.5 (15.5)	18.1 (22.8)	6.1 (59.7)	9.4 (45.3)

*ADC* antibody–drug conjugate, *AUC* area under the curve, *AUC*_*(0-inf)*_ AUC from time zero to infinity, *AUC*_*(0-tau)*_ AUC for one dosing interval, *AUC*_*(0-tlast)*_ AUC up to last quantifiable study time-point, *C*_*EOI*_ concentration at end of infusion, *CL* clearance, *C*_*max*_ maximum observed concentration, *CVb* between-subject coefficient of variation, *h* hour, *inf* infinity, *t*_*½*_ terminal phase half-life (single dose), *t*_*last*_ last time point where the concentration is above the limit of quantification, *t*_*max*_ time to C_max_, *Vss* volume of distribution at steady state

^a^Median (range)

^b^Individual values presented as only two patient samples assessed

^c^Caution should be used in interpreting the t_½_ results as Lambda Z interval < 2 × estimated t_½_ in 1 of 3 (Arm A, ADC), 3 of 3 (Arm A, total antibody) and 2 of 4 (Arm B, ADC and total antibody) patients

**Table 6:** Summary of derived cys-mcMMAF PK parameters during Cycle 1 (PK population)

Geometric mean, (% CVb)	Arm A Belantamab mafodotin + bortezomib/dexamethasone (N=3)	Arm B Belantamab mafodotin + pomalidomide/dexamethasone (N=4)
C_max_(pg/mL)	857.6 (33.0)	905.4 (28.4)
t_max_ (h)^a^	22.4 (22.1–23.1)	23.0 (22.0–24.2)
t_last_ (day)^a^	6.9 (6.8–7.0)	6.9 (3.9–7.8)
AUC_(0-tlast)_(h*ng/mL)	80.1 (22.5)	82.6 (42.3)

*AUC*_*(0-tlast)*_ area under the curve up to the last quantifiable study time-point, *C*_*max*_ maximum observed concentration, *CVb* between-subject coefficient of variation, *cys-mcMMAF* cysteine maleimidocaproyl monomethyl auristatin F, *h* hours, *t*_*last*_ last time point where the concentration is above the limit of quantification, *t*_*max*_ time to C_max_

^a^Median (range)

## Discussion

DREAMM-11 was a Phase 1, open-label study of belantamab mafodotin monotherapy (Part 1) [11], and combination therapy (Part 2) in patients with RRMM. This was the first study to investigate the tolerability, safety, efficacy and PK of belantamab mafodotin alone and in combination with the SoC regimens bortezomib plus dexamethasone and pomalidomide plus dexamethasone, specifically in a Japanese patient population. In Part 1, both doses of belantamab mafodotin monotherapy were tolerated [11]. Similarly, in Arm A of Part 2, the combination of belantamab mafodotin with bortezomib plus dexamethasone was also tolerated, with no DLTs reported. Hepatoxicity is known to be associated with pomalidomide treatment [13], and, in the four patients who received belantamab mafodotin in combination with pomalidomide plus dexamethasone, one DLT of liver injury was reported 7 days post-first dose; it was not deemed to be serious and resolved after discontinuation of treatment. According to the 3 + 3 study design for assessing tolerability, the occurrence of a DLT in the first three patients of Arm B required enrolment of an additional three patients. However, as only four patients were enrolled in Arm B, it is not possible to draw any firm conclusions on the tolerability of belantamab mafodotin in combination with pomalidomide and dexamethasone. Nevertheless, a comprehensive safety review of available data determined that no safety concerns were specifically expected with this treatment combination in a Japanese patient population. Similar to Part 1, where no new safety signals for belantamab mafodotin monotherapy were identified in Japanese patients compared with Western patients [6, 11, 14], no new safety signals in Japanese patients were identified with the combinations investigated in Part 2 of the study [7, 8]. Furthermore, the safety profile of each combination was consistent with those of the individual agents [13, 15]. All patients experienced non-serious Grade ≥ 3 AEs; the most common of these across the two treatment arms were thrombocytopenia, lymphopenia and decreased neutrophil counts. Only one SAE (COVID-19 – not related to study treatment) was reported across both treatment arms. Corneal events of a maximum of Grade 3 (based on the protocol-defined scale for keratopathy and visual acuity) were reported in all patients; these were managed with dose delays or interruptions, particularly in patients who had a long duration of treatment and did not require treatment withdrawal, and resolved after a median of 127 days in Arm A and 78 days in Arm B.

In DREAMM-11 Part 1, ORR with belantamab mafodotin 2.5 mg/kg monotherapy was 50% (2/4 patients) [11]. In Part 2, clinical activity was demonstrated with both combination treatments investigated; all three patients (100%) treated with belantamab mafodotin plus bortezomib and dexamethasone in Arm A achieved an overall response. These findings align with those from the recent DREAMM-7 trial that demonstrated increased PFS, extended time to progression or death, and a clinically meaningful OS trend compared with current SoC (daratumumab plus bortezomib and dexamethasone), in patients with RRMM [7]. All patients in Arm A of the present study demonstrated a deep response (VGPR) to treatment, and two of these responders had a long treatment duration, continuing to receive belantamab mafodotin after the data cut-off date. In Arm B, two of the four patients (50%) treated with belantamab mafodotin plus pomalidomide and dexamethasone achieved an overall response, with one patient achieving a deep response (CR) and the other achieving a PR; both of whom had a long treatment duration. The patient who experienced a DLT during Cycle 1 had stable disease at the time of discontinuation, while the remaining patient withdrew from treatment prior to Cycle 2, due to progressive disease.

Importantly, responses to either of the belantamab mafodotin combination treatments in Arm A and B were sustained despite any dose delays or interruptions. This finding is aligned with the ALGONQUIN study that recently reported an ORR of 89.8% across all doses of belantamab mafodotin in combination with pomalidomide and dexamethasone [8]. Belantamab mafodotin (2.5 mg/kg Q4W for Cycle 1, followed by 1.9 mg/kg for Cycle 2 onwards) in combination with pomalidomide and dexamethasone is also being investigated in the ongoing Phase 3 multicenter trial, DREAMM-8 where ORR was 77% [9]. In DREAMM-8, a statistically significant PFS benefit compared with pomalidomide plus bortezomib and dexamethasone (PVd) as well as a positive trend in OS, and a safety profile that is broadly consistent with the individual agents [9].

While exposure parameters for belantamab mafodotin were slightly higher with the combinations assessed in Part 2 than with belantamab mafodotin monotherapy in Part 1, this was likely due to the small sample size and variability of disease burden between patients in each part of the study. Importantly, PK profiles were as expected based on data obtained for Western patient populations.

A major limitation of the study is the small number of patients included. Nonetheless, Part 2 of this study built on the monotherapy data from Part 1, demonstrating that the combination of belantamab mafodotin with bortezomib/pomalidomide plus dexamethasone led to clinical activity in Japanese patients with RRMM. This study has provided support for the inclusion of Japanese patients in the larger, global, Phase 3 trials of belantamab mafodotin combinations.

## Supplementary Information

Below is the link to the electronic supplementary material.

Supplementary file1 (PDF 335 KB)

## Data Availability

Please refer to GSK weblink to access GSK’s data sharing policies and as applicable seek anonymized patient level data via the link https://www.gsk-studyregister.com/en/**.**
