# Supplementary material for: DREAMM-11, Part 2: Japanese phase I trial of belantamab mafodotin combination therapies in relapsed/refractory multiple myeloma
Source: Int J Hematol. 2024 Dec 24;121(2):174–86. doi: 10.1007/s12185-024-03889-8 (PMC11782446; doi:10.1007/s12185-024-03889-8)
Supplement: Supplementary file 1 — Supplementary file1 (PDF 335 KB) [file 12185_2024_3889_MOESM1_ESM.pdf]

Supplementary Materials

DREAMM-11, Part 2: Japanese Phase I Trial of Belantamab Mafodotin Combinations in Relapsed/Refractory Multiple Myeloma

Kazutaka Sunami *et al.*

Supplementary Section 1: DREAMM-11 Part 2 eligibility criteria

Supplementary Figure 1: DREAMM-11 study design

Supplementary Table 1: Protocol-defined scale for keratopathy and visual acuity

Supplementary Table 2: Summary of dose delays and dose reductions (all treated population)

Supplementary Table 3: Adverse events of special interest (all treated population)

Supplementary Section 1: DREAMM-11 Part 2 eligibility criteria

Inclusion criteria

Patients were eligible to be included in the clinical study only if all of the following criteria were met:

1. Patient provided signed written informed consent, which included compliance with the requirements and restrictions listed in the consent form
2. Male or female, ≥20-years of age at the time consent was obtained
3. ECOG performance status of 0–2
4. Histologically or cytologically confirmed diagnosis of multiple myeloma (MM), as defined according to International Myeloma Working Group (IMWG) 2014 criteria [Rajkumar, 2014], in patients who fulfilled all of the following:
    - Had undergone stem-cell transplant, or were considered transplant ineligible
    - Had received ≥1 prior line of antimyeloma therapies
    - Had documented disease progression during or after their most recent therapy
5. Had measurable disease with at least one of the following:
    - Serum M-protein ≥0.5 g/dL (≥5 g/L)
    - Urine M-protein ≥200 mg/24 hours
    - Serum free light chain (FLC) assay: involved FLC level ≥10 mg/dL (≥100 mg/L) and an abnormal serum FLC ratio (<0.26 or >1.65)
6. Patients with a history of autologous stem cell transplant (ASCT) were eligible for study participation provided the following eligibility criteria were met:
    - Transplant was >100 days prior to study enrolment.
    - No active infection
7. **Female patients:** contraceptive use by women had to be consistent with local regulations regarding the methods of contraception for those participating in clinical studies
    - A female patient was eligible to participate if she was not pregnant or breast feeding, and at least one of the following conditions applied:
        - Was not a woman of childbearing potential (WOCBP)

- **Arm A:** was a WOCBP and using a contraceptive method with a failure rate <1% per year, preferably with low user dependency, during the treatment period and for 4 months after the last dose of belantamab mafodotin, and 7 months from the last dose of bortezomib and agreed not to donate eggs (ova, oocytes) for the purpose of reproduction during this period. The investigator had to evaluate the effectiveness of the contraceptive method in relationship to the first dose of study intervention. A WOCBP needed to have a negative highly sensitive serum pregnancy test (as required by local regulations) ≤72 hours before the first dose of study intervention and agree to use effective contraception during the clinical study and for 4 months after the last dose of belantamab mafodotin, and 7 months from the last dose of bortezomib.
- **Arm B:** Due to pomalidomide being a thalidomide analogue with risk for embryo-foetal toxicity and prescribed under a restricted distribution program, WOCBP patients were eligible if they committed either to abstain continuously from heterosexual sexual intercourse or to use 2 methods of reliable birth control, beginning 4 weeks prior to initiating treatment with pomalidomide, during therapy, during dose interruptions and continuing for 4 weeks following discontinuation of pomalidomide treatment. Thereafter, WOCBP patients had to use a contraceptive method with a failure rate <1% per year for a further 3 months, and agree not to donate eggs (ova, oocytes) for the purpose of reproduction during this period. Two negative pregnancy tests had to be obtained prior to initiating pomalidomide therapy. The first test had to be performed within 10 to 14 days and the second test within 24 hours prior to prescribing pomalidomide therapy. The investigator was responsible for review of medical history, menstrual history, and recent sexual activity to decrease the risk for inclusion of a woman with an early undetected pregnancy.

8. **Male patients:** Contraceptive use by men had to be consistent with local regulations regarding the methods of contraception for those participating in clinical studies. Male patients were eligible to participate if they agreed to the following from the time of first dose of study intervention until 6 months after the last dose of belantamab mafodotin, 4 months after the last dose of bortezomib (only Arm A), and 4 weeks after the last dose of pomalidomide (only Arm B) to allow for clearance of any altered sperm:
    - To refrain from donating sperm

      PLUS either:
    - To be abstinent from heterosexual intercourse as their preferred and usual lifestyle (abstinent on a long term and persistent basis) and agreed to remain abstinent

      OR
    - Agreed to use contraception/barrier as follows: Agreed to use a male condom, even if they had undergone a successful vasectomy, and female partner to use an additional contraceptive method with a failure rate <1% per year, when having sexual intercourse with a WOCBP (including pregnant females)
9. All prior treatment-related toxicities (defined by NCI-CTCAE, version 4.03), had to be Grade ≤1 at the time of enrolment, except for alopecia. Patients with Grade 2 peripheral neuropathy could be enrolled into Arm B but not into Arm A
10. Adequate organ system functions

Exclusion criteria

Patients were excluded from the clinical study if any of the following criteria were met:

1. Systemic antitumor therapy within 14 days, or plasmapheresis within 7 days prior to the first dose of study intervention
2. Symptomatic amyloidosis, active 'polyneuropathy, organomegaly, endocrinopathy, myeloma protein, and skin changes' syndrome, active plasma cell leukemia at the time of screening

3. Use of an investigational drug within 14 days or 5 half-lives, whichever was shorter, preceding the first dose of study intervention. Prior treatment with a monoclonal antibody (mAb) within 30 days of receiving the first dose of study intervention. Prior B cell maturation antigen (BCMA) targeted therapy
4. History of an allogeneic stem-cell transplant
5. Current use of prohibited medications/device or planned use of any of these during the study period
6. Current corneal epithelial disease except mild punctate keratopathy
7. Presence of active renal condition (infection, requirement for dialysis or any other condition that could affect patient’s safety). Patients with isolated proteinuria resulting from MM were eligible, provided they fulfilled prespecified criteria
8. Evidence of active mucosal or internal bleeding
9. Any major surgery ≤4 weeks prior to screening
10. Any serious and/or unstable pre-existing medical, psychiatric disorder, or other conditions (including laboratory abnormalities) that could interfere with patient’s safety, obtaining informed consent or compliance to the study procedures
11. Active infection requiring treatment (antibiotic, antiviral, or antifungal treatment)
12. Evidence of severe or uncontrolled systemic diseases
13. Malignancies other than the disease under clinical study were excluded, except for any other malignancy from which the patient had been disease-free for >2 years and, in the opinion of the investigators and medical monitor, did not affect the evaluation of the effects of this study intervention on the currently targeted malignancy (MM)
14. Evidence of cardiovascular risk including any of the following: Corrected QT interval Fridericia (QTcF) ≥470 msec (the QT interval values had to be corrected for heart rate by Fridericia’s formula); Evidence of current clinically significant uncontrolled arrhythmias,

including clinically significant electrocardiogram abnormalities such as second degree (Type II) or third degree atrioventricular block; history of myocardial infarction, acute coronary syndromes (including unstable angina), coronary angioplasty, or stenting or bypass grafting within 6 months of Screening; Class III or IV heart failure as defined by the New York Heart Association functional classification system; uncontrolled hypertension

15. Known immediate or delayed hypersensitivity reaction or idiosyncrasy to drugs chemically related to belantamab mafodotin or any of the components of the study intervention
16. Pregnant or lactating female or female who was interrupting lactation
17. Known human immunodeficiency virus infection
18. Patients with Hepatitis B were excluded unless prespecified criteria could be met
19. Positive HCV antibody test result or positive HCV RNA test result at screening or ≤3 months prior to first dose of study intervention. Note: Patients with positive HCV antibody due to prior resolved disease could only be enrolled if a confirmatory negative HCV RNA test was obtained; Hepatitis RNA testing was optional and patients with negative HCV antibody tests were not required to also undergo HCV RNA testing
20. Current unstable liver or biliary disease per investigator assessment, defined by the presence of ascites, encephalopathy, coagulopathy, hypoalbuminemia, esophageal or gastric varices, persistent jaundice, or cirrhosis. Note: Stable chronic liver disease (including Gilbert's syndrome or asymptomatic gallstones) or hepatobiliary involvement of malignancy was acceptable if patient otherwise met the entry criteria
21. Previously diagnosed with interstitial lung disease or current complication of interstitial lung disease

Reference

Rajkumar SV, Dimopoulos MA, Palumbo A, et al. International Myeloma Working Group updated criteria for the diagnosis of multiple myeloma. Lancet Oncol. 2014;15(12):e538-48.

Supplementary Figure 1: DREAMM-11 study design

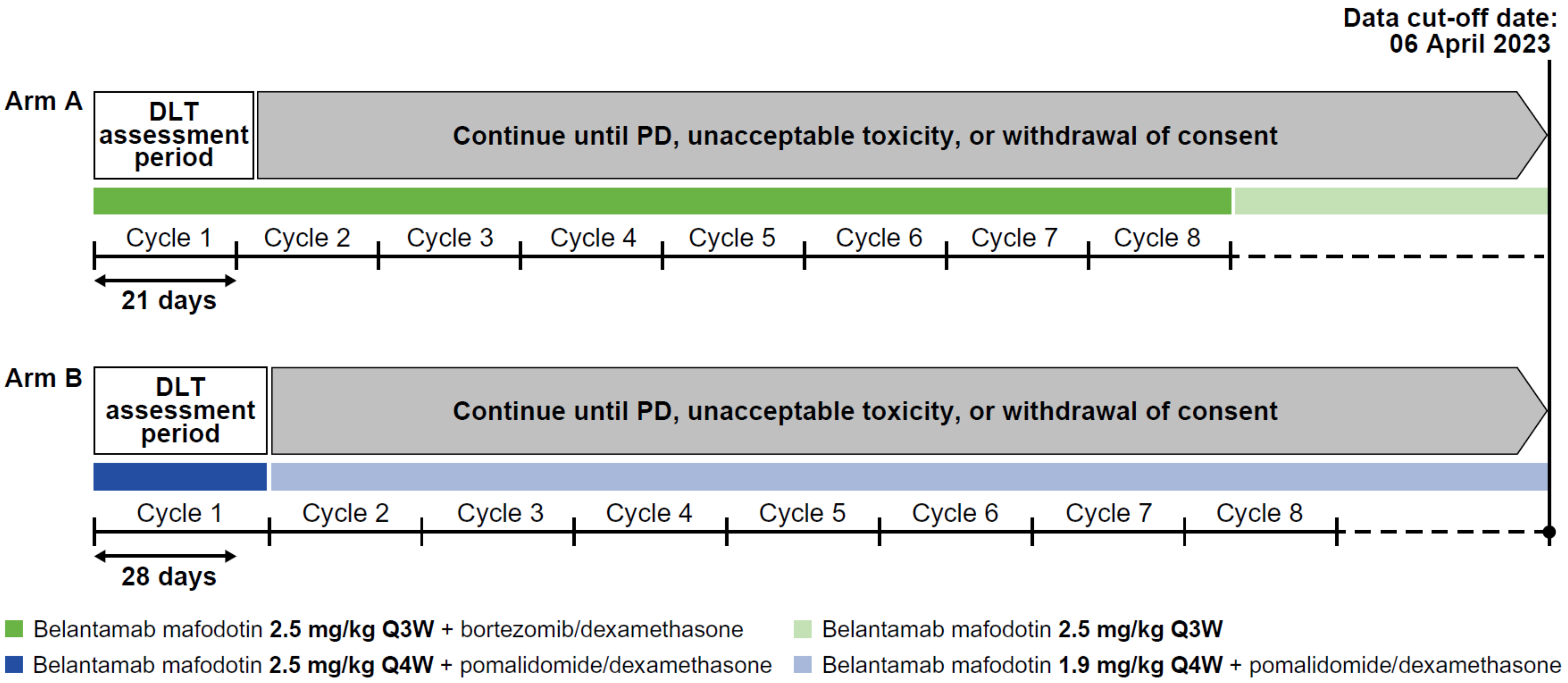

This figure was adapted from Iida S, et al. 49th Annual Meeting of the Japanese Society of Myeloma, May 31–June 1, 2024 and has been reused with permission.

DLT, dose-limiting toxicity; PD, progressive disease; Q#W, every # weeks.

Supplementary Table 1: Protocol-defined scale for keratopathy and visual acuity[a]

Measure	Grade 1	Grade 2	Grade 3	Grade 4
Ophthalmic exam finding	Mild superficial keratopathy (change from baseline)	Moderate punctate keratopathy and/or mild/patchy microcysts and/or mild/patchy epithelial or stromal edema and/or sub-epithelial haze (peripheral) and/or active stromal opacity (peripheral)	Severe punctate keratopathy and/or diffuse microcysts and/or diffuse epithelial or stromal edema and/or sub-epithelial haze (central) and/or active stromal opacity (central)	Corneal ulcer

Visual Acuity[b]	Change of 1 line from baseline	Change of 2–3 lines from baseline and not worse than 20/200[c]	Change of more than 3 lines from baseline and not worse than 20/200[c]	Worse than Vision 20/200[c]

[a]Grading was based on most severe finding; if eyes differed in severity, grading using the protocol-defined scale should be based on the more severe eye; [b]change in visual acuity due to belantamab mafodotin-associated ocular events; if change in vision was for a different reason, grading was driven by ophthalmic exam findings; [c]change in visual acuity by Snellen chart; if a patient had a baseline visual acuity of 20/200 or worse in an eye, ophthalmic exam findings drove the event grading.

Supplementary Table 2. Summary of dose delays and dose reductions (all treated population)

	**Arm A** **Belantamab mafodotin + bortezomib/dexamethasone (N=3)**	**Arm B** **Belantamab mafodotin + pomalidomide/dexamethasone (N=4)**
**Belantamab mafodotin dose reductions**		
Participants with any dose reduction, n (%)	2 (67)	N/A
Total number of dose reductions	2	N/A
Number of dose reductions, n (%)		
0	1 (33)	N/A
1	2 (67)	N/A
Reasons for reduction[a], n (%)		
Corneal exam findings	2 (100)	N/A
Participants with dose reductions by dose, n (%)		
1st dose	0/3	N/A
2nd dose	2/3 (67)	N/A
**Belantamab mafodotin dose delays**[†]		

Participants with any dose delay, n (%)	3 (100)	2 (50)
Total number of dose delays	12	9
Time (days) to first delay, n (%)		
Arm A: >63<br>Arm B: >84	3 (100)	2 (100)
Mean days (SD)	114.0 (51.47)	99.0 (19.80)
Median days (range)	106.0 (67–169)	99.0 (85–113)
Number of dose delays		
1	1 (33)	0
≥3	2 (67)	2 (50)
Duration (days) of delays, n (%)		
Arm A: 22–42<br>Arm B: 29–56	0	3 (33)
Arm A: >42<br>Arm B: >56	12 (100)	6 (67)
Reasons for delay, n (%)		
Corneal exam findings	12 (100)	7 (78)
Other AE	0 (0)	2 (22)

Number of participants with dose delays by dose, n (%)		
1st dose	0/3	0/4
2nd dose	3/3 (100)	1/2 (50)
3rd dose	2/3 (67)	2/2 (100)
4th dose	2/2 (100)	2/2 (100)
5th dose	2/2 (100)	2/2 (100)
6th dose	1/1 (100)	2/2 (100)
7th dose	1/1 (100)	N/A
8th dose	1/1 (100)	N/A

Dose reductions were not permitted in Arm B.

[a]Patients may be counted multiple times in the same "reason" row, if the patient had multiple reductions for the same reason; [†]dose delays resulted from a dose hold which was followed by a subsequent administration of belantamab mafodotin.

AE, adverse event; SD, standard deviation.

Supplementary Table 3: Adverse events of special interest (all treated population)

	**Arm A**<br><br>**Belantamab mafodotin + bortezomib/dexamethasone**<br><br>**(N=3)**	**Arm B**<br><br>**Belantamab mafodotin + pomalidomide/dexamethasone**<br><br>**(N=4)**
**Any corneal event (protocol-defined scale for keratopathy and visual acuity), n (%)**	3 (100)	4 (100)
Maximum Grade		
Grade 3, n (%)	3 (100)	4 (100)
Time of event onset, days, n (%)		
Arm A: 1–21<br>Arm B: 1–28	0 (0)	0
Arm A: 22–42<br>Arm B: 29–56	3 (100)	3 (75)
Arm A: 43–63<br>Arm B: 57–84	0 (0)	1 (25)
Arm A: 64–105<br>Arm B: 85–112	0 (0)	0 (0)
Arm A: >105	0 (0)	0 (0)

Arm B: >113		
Mean (SD) days	23.0 (1.7)	36.0 (14.0)
Median (range) days	22 (22–25)	29 (29–57)
Duration of event, days, n (%)		
Arm A: 1–21<br>Arm B: 1–28	0	0
Arm A: 22–42<br>Arm B: 29–56	0	0
Arm A: >42<br>Arm B: >56	3 (100)	4 (100)
Mean (SD) days	155.0 (87.43)	102.5 (64.0)
Median (range) days	127.0 (85–253)	78 (57–197)
**Any thrombocytopenic event, n (%)**	3 (100)	4 (100)
Thrombocytopenia	3 (100)	4 (100)
**Infusion-related reactions, n (%)**	0 (0)	0 (0)

[a]Includes thrombocytopenia and platelet count decreased which were not reported for the same patients.

SD, standard deviation.
